# Effects of the surface processing on the tribological performance of C/SiCs under dry friction

**DOI:** 10.1038/s41598-020-62914-y

**Published:** 2020-04-06

**Authors:** Bin Lin, Jinhua Wei, Tianyi Sui, Haoji Wang

**Affiliations:** Key Laboratory of Advanced Ceramics and Machining Technology of Ministry of Education, Tianjin University, Tianjin, China

**Keywords:** Mechanical engineering, Structural materials

## Abstract

A new friction counterpart for carbon fiber-reinforced silicon carbide ceramic-matrix composites (C/SiCs) and zirconia (ZrO_2_) toughened by magnesia ceramics is proposed. The effects of the C/SiC surface processing parameters friction on the tribological performance are investigated under dry friction and ambient temperature conditions. The wear tests are carried out using the pin-on-disc friction method. Scanning electron microscopy (SEM) on an instrument equipped with an energy dispersive spectroscopy (EDS) is used to observe the surfaces of the pins and discs before and after the application of friction to reveal the wear mechanism. The results show that surface processing influenced the tribological properties of C/SiC significantly. When the pressure is 30 N, the speed is 0.5 m/s, and the C/SiC surface is ground using 1500# sandpaper, the counterpart tribological performance is the best among the samples considered herein. It is found that the retention ability of the counterparts influenced the tribology performance significantly.

## Introduction

The rapid technological development in the aerospace field has resulted in increasingly severe working conditions for structural components^1,2^. Traditional materials are no longer suitable for increasingly harsh environments, such as those with high temperatures, sharp impacts, extreme corrosion, and severe wear^3,4^. Thus, alternative materials are required to meet the needs of these applications.

Fiber-reinforced ceramic-matrix composites (FRCMCs) have promising applications in the aerospace field owing to their good adaptability and stability in harsh environments^5^. They not only have an improved toughness because of the reinforcing fibers and a high hardness and excellent temperature resistance owing to the ceramic matrix but also have other excellent properties (e.g., corrosion and wear resistance, good thermal capacity and dimensional stability, light weight, and insensitivity to cracks) due to the synergistic effect of the fibers and matrix^6–10^. Among the FRCMCs, carbon fiber-reinforced silicon carbide ceramic-matrix composites (C/SiCs) have received special attention because of their excellent temperature and wear resistance. Carbon fibers provide good lubrication; in addition, SiC ceramics react readily with the air, producing a silica (SiO_2_) protection film^11,12^ and improving the wear resistance of the C/SiC, which increases its attractiveness in the field of tribology. To date, C/SiC is a preferred material for internal contact friction pairs in aero engines^9^. Zirconia (ZrO_2_) toughened by magnesia (MgO) ceramic also has good stability, high hardness and strength, and excellent wear and temperature resistance^13,14^. Accordingly, it is assumed that a friction pair composed of C/SiC and ZrO_2_ has excellent high temperature and wear resistance. However, the friction and wear behaviors of the counterpart lack further study, resulting in an insufficient understanding of the tribological mechanisms. During the friction process, the surface characteristics of the contact plateaus^15–17^, the working conditions^18–20^, and the external environment^21–25^ play important roles on the tribological performance. Especially, the surface processing, influencing the surface quality of the friction pair, could affect the tribology properties of the friction pair significantly^26–28^. Thus, it is very important to investigate the influence of surface processing on the tribology properties of C/SiC and ZrO_2_ pair. In the present work, the effects of the surface processing parameters of the C/SiC pins with a 90 deg fiber orientation during the friction process are studied. The friction style of the pin-on-disc method and the experimental conditions of ambient temperature and dry friction are used. The aim is to reveal the friction and wear mechanisms of this counterpart in detail to expand its application field.

## Results and discussion

### Surface processing of the C/SiCs on the tribological performance of the counterpart

**Figure 1:**
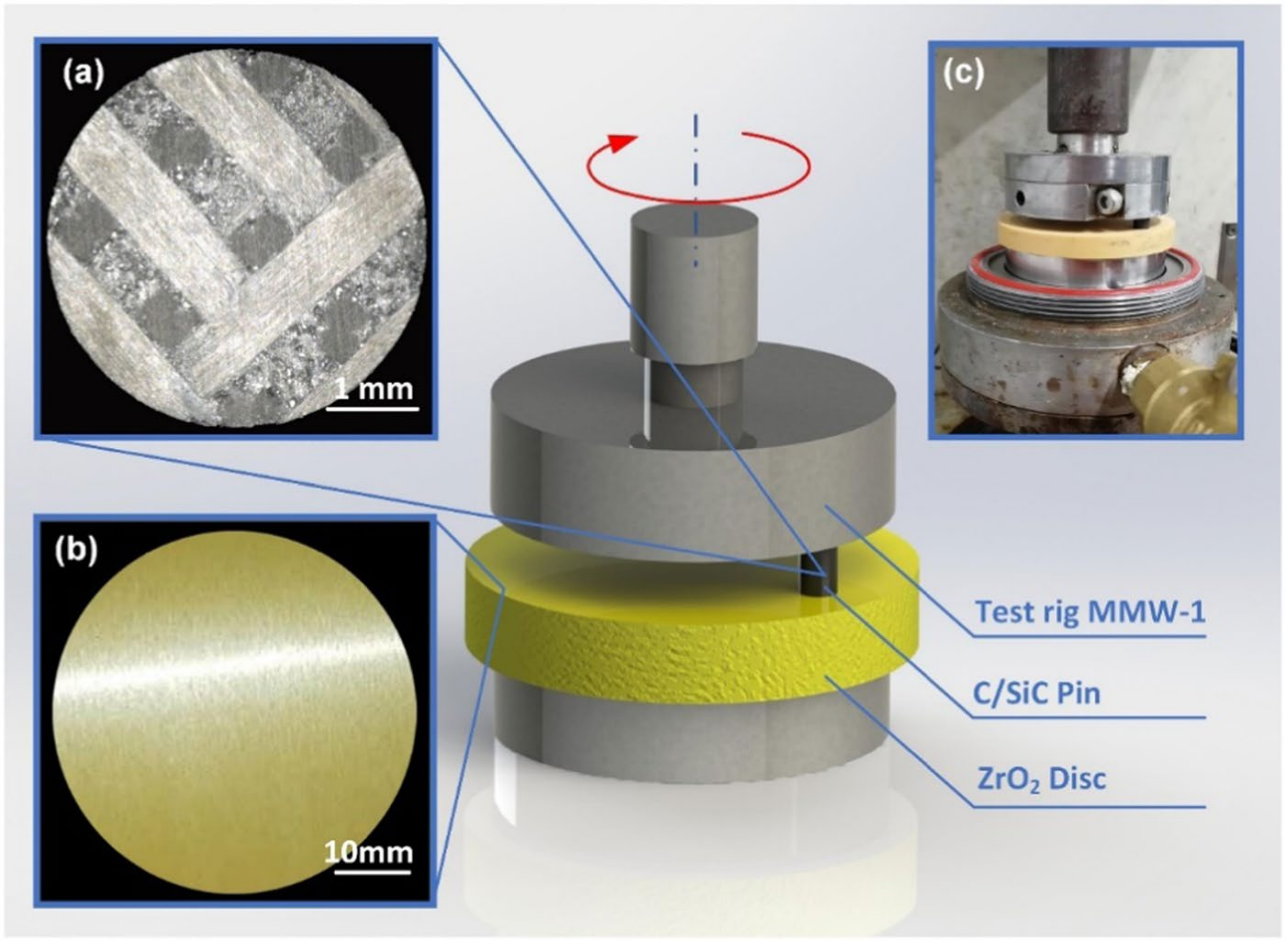
Experimental materials and apparatus: (**a**) C/SiC pin surface, (**b**) ZrO_2_ disc surface, and (**c**) experimental apparatus.

The surfaces of the C/SiC and ZrO_2_ and the experimental setup are shown in Fig. 1. All tests were repeated for three times and named as 1rd, 2nd and 3rd. Both the average COF (Ave. COF) and maximum COF (Max. COF) were recorded. The Ave. COF and error bar were the average value and variability of three times friction coefficient of 7200 s tribology test. The Max. COF is the highest COF recorded during the friction test.

**Figure 2:**
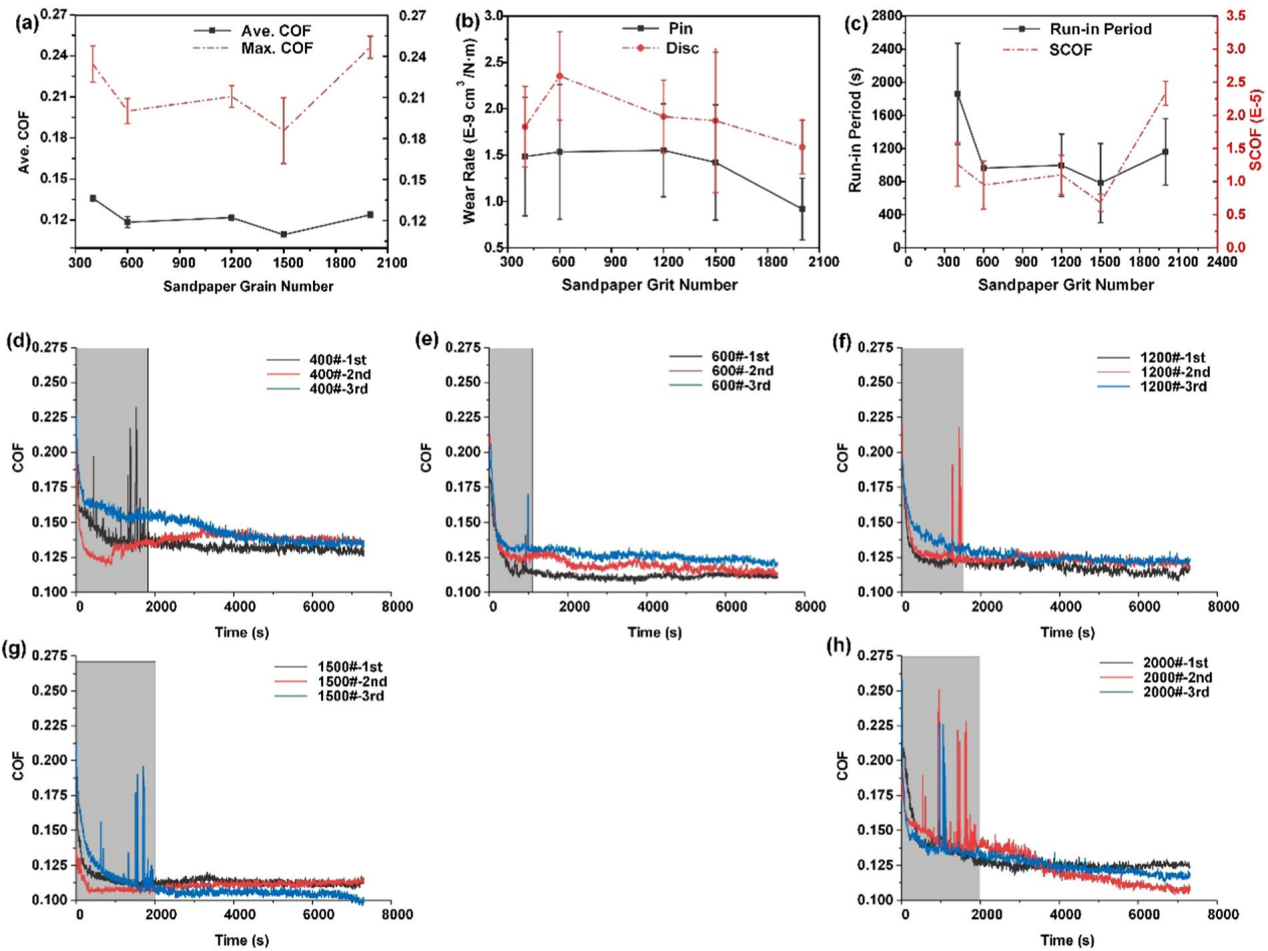
Friction experiment results as a function of sandpaper grit: (**a**) Ave. COF and Max COF, (**b**) wear rate of the pin and disc, and (**c**) time entering the run-in period and SCOF. (**d–h**) COFs of 400#, 600#, 1200#, 1500# and 2000# sandpapers, respectively.

Figure 2 shows the friction experiment results as a function of the sandpaper grit size. It shows that the Ave. COF and the Max. COF have nearly the same trends (Fig. 2(a)). The Max COF occurs during the initial period of a friction process when the counterpart has not yet entered a stable run-in period, while the Ave. COF is an indicator of the stable run-in period. The reduction in the surface roughness of the contact interface with friction process reduced the COF significantly during the friction test^29^. In addition, when the 1500 sandpaper is used, both Ave. COF and Max COF are the smallest, with values of 0.110 and 0.186, respectively. The same situation happens during the time entering the run-in period and with the standard deviation of the Ave. COF (SCOF) (Fig. 2(c)), which means that the initial run-in period is the shortest and the friction process is the most stable when a 1500 grit sandpaper is used. As Fig. 2(b) shows, although the wear rates of the pin and disc present a nearly downward trend with an increase in the sandpaper grit size, their values do not change substantially. Additionally, from Fig. 2(d–h), we can easily see that the COF for the 1500# sandpaper is more stable and obviously lower than that of the other sandpapers. Therefore, when the C/SiC surface is ground using 1500# sandpaper, the counterpart has the best tribological performance of the samples studied herein. It should be noted here that the C/SiC–ZrO_2_ counterpart shows excellent tribology performance without lubrication (optimal COF is 0.11), which is much lower than self-mated C/SiC and almost the same of PTFE and PEEK materials^30–33^.

The surface roughness of the ZrO_2_ pins before and after tribology test is shown in Table 1. It could be found that the surface of C/SiC changed significantly when ground with sandpapers of different sizes. The surface morphologies of C/SiCs surfaces of different sandpaper sizes are studied in our previous study and polishing scratches could be found on pin surface^34^. However, after the tribology test, the pin surface became ultra-smooth, which could be attributed to wear and filling of wear debris.

**Table 1:** Surface roughness of C_f_/SiC pins before and after tribology test.

Ave. Sa (μm)	400#	600#	1200#	1500#	2000#
Before test	35.6	28.8	14.2	2.1	7.5
After test	0.8	0.7	0.7	0.6	0.7

**Figure 3:**
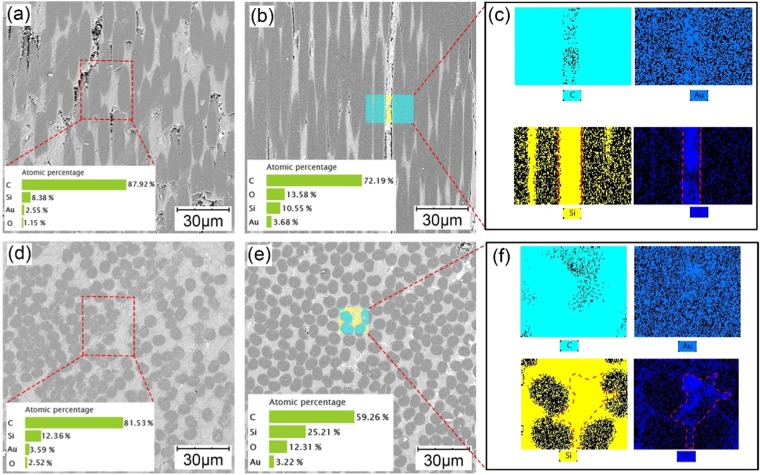
SEM images and EDS analyses of the C/SiC pin surface before and after the friction process: (**a,d**) side and end surfaces of a fiber bundle before friction, (**b,e**) side and end surfaces of a fiber bundle after friction, and (**c,f**) EDS results of the highlighted areas in (**b,e**).

The SEM image and EDS analysis of the surface of the pin ground with the 1500# sandpaper are shown in Fig. 3. The 90 deg C/SiC surface is composed of two fiber bundle orientations based on the location of the side and end surfaces, and there is a minor amount of matrix material surrounding the fibers or filling the voids. Thus, the surfaces of the two fiber bundle orientations are analyzed. The grinding scratches are clearly visible on both surfaces before the friction process (Fig. 3(a,d)); moreover, there is a smaller amount of oxygen element (O) before the friction process than that after it. However, the grinding scratches disappear after the friction process (Fig. 3(b,e)) and large amounts of O appear, especially where the silicon (Si) exists (Fig. 3(c,f)). This could be due to the adsorption of ZrO_2_ wear debris.

**Figure 4:**
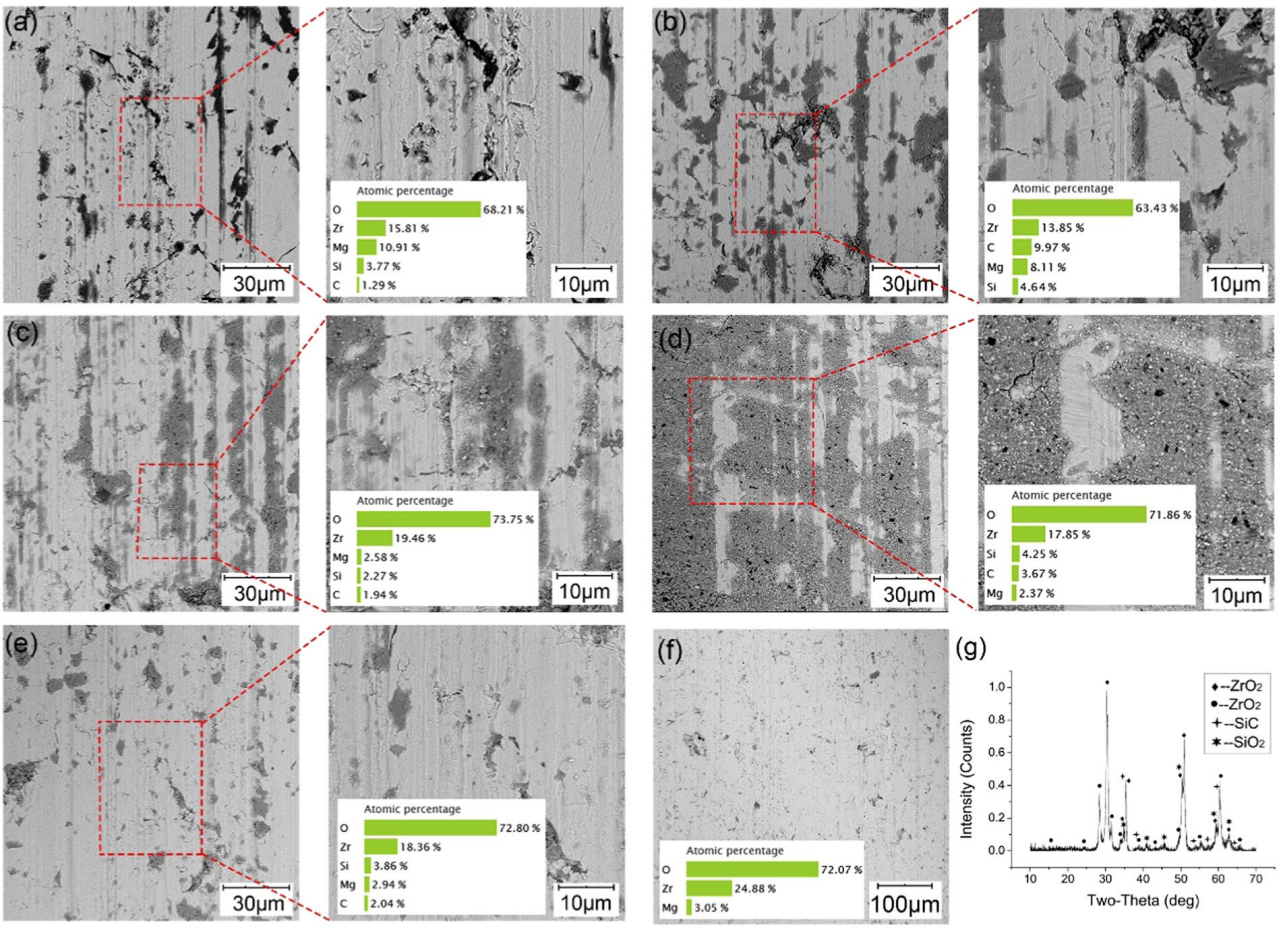
SEM images, EDS and XRD analyses of the ZrO_2_ disc surface before and after friction: (**a**) after grinding with 400# sandpaper and after friction, (**b**) after grinding with 600# sandpaper and after friction, (**c**) after grinding with 1200# sandpaper and after friction, (**d**) after grinding with 1500# sandpaper and after friction, (**e**) after grinding with 2000# sandpaper and after friction, (**f**) disc surface before the friction process, and (**g**) XRD analysis of the ZrO_2_ surface after the friction process.

As Fig. 4(a–e) shows, the friction scratches are quite visible along the friction direction after the experiments, and some are filled with dark gray materials on the ZrO_2_ disc compared with the shallow processing traces before the friction process (Fig. 4(f)). The disc surface contains extra elements of carbon (C) and Si that are not initially present in addition to zirconium (Zr), magnesium (Mg) and O through the EDS analyses. This phenomenon reflects that the surface asperities of the C/SiC are worn into wear debris, some of them take part in the friction process, and some are embedded in the disc surface. However, the area of the embedded substances changes dramatically with the sandpaper size. The disc surfaces ground with the 400# and 2000# sandpapers have the smallest area of embedded substances, the next up is the surfaces ground with the 600# and 1200# sandpapers, and the surface ground with the 1500# sandpaper has the largest area. Compared with Fig. 2, the embedded area trend is almost the same as that of the COF trend. The larger the area is, the smaller the COF, the more stable the friction process, and the shorter the initial run-in period. That is, the embedded substances form an intermittent or even continuous film on the disc surface, which has a lubricating effect. The C/SiC surface includes O before and after the friction process, and the amount of O only changes. To investigate the surface change during the friction process, the ZrO_2_ disc surface after the friction process with C/SiC ground by 1500# sandpaper is analyzed with X-ray diffraction (XRD). As Fig. 4(g) shows, there are two phases of ZrO_2_, SiC and SiO_2_ on the disc surface after the friction process, which means wear debris adsorb on both counterparts.

### Wear mechanism of the surface processing of the C/SiCs

**Figure 5:**
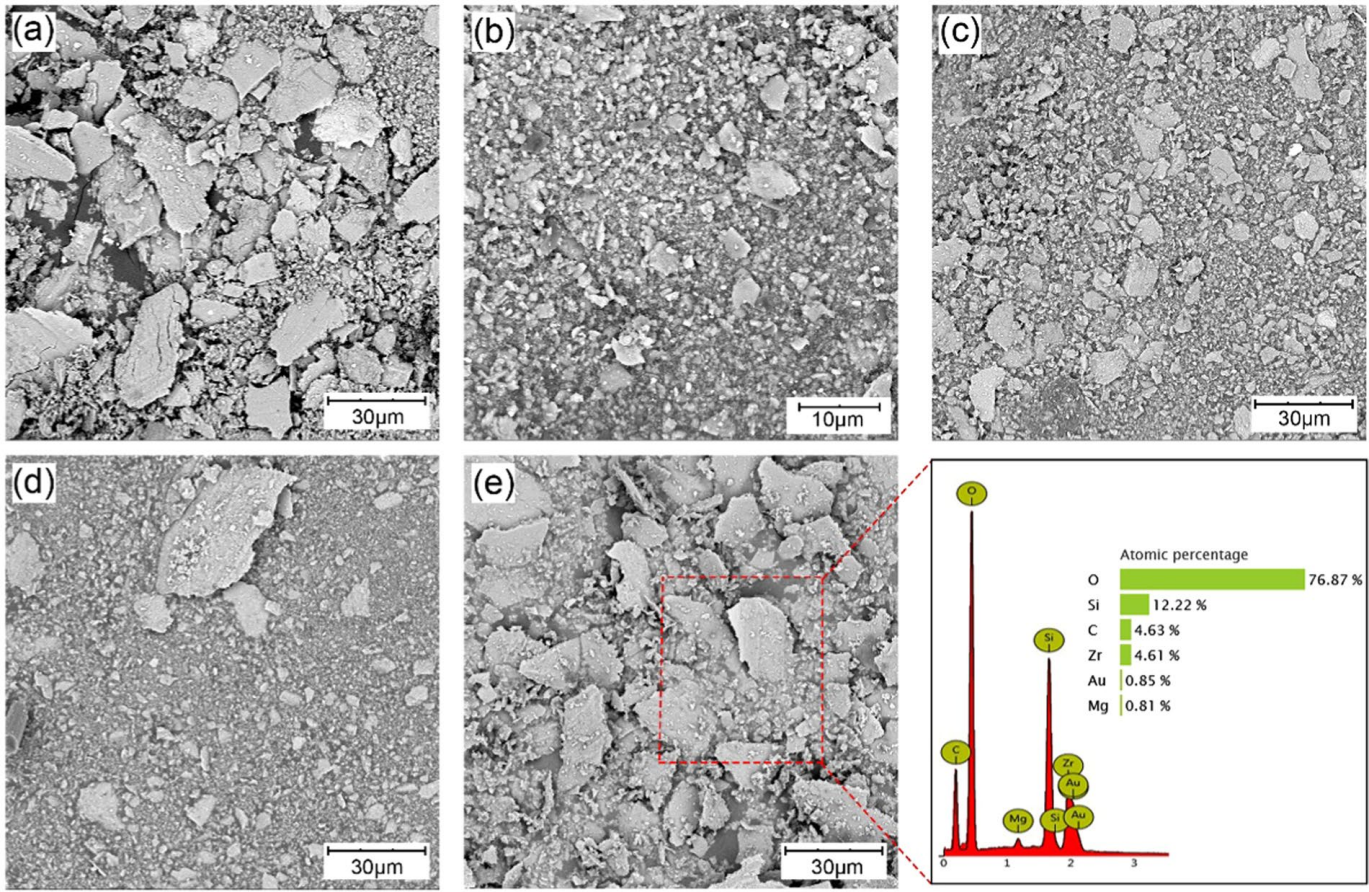
SEM images of wear debris from different sandpaper sizes: (**a**) 400#, (**b**) 600#, (**c**) 1200#, (**d**) 1500#, and (**e**) 2000#. (**f**) EDS analysis of (**e**).

According to the experimental results and analyses above, when other conditions are held constant and only the C/SiC surface processing parameters are changed, the factor that influences the tribological performance is the formation of lubrication film, which is due to the combined action of the cutting ability of the C/SiC pin on the ZrO_2_ disc and the retention ability of the disc to the wear debris. C/SiC with rough surface will cut the ZrO_2_ disc, cause wear and generate grooves which could the retain the wear debris. The C/SiC pin would be polished and filled with wear debris at the same time. The debris contains all elements of the pin and disc, especially Si and O (Fig. 5(f)). The debris size is quantitatively analyzed. 50 debris of each sample were chosen randomly from SEM photos and the longest size of the debris was taken as the data for statistics. The column graph of the statistic result is shown in Figure S1 in Supporting Information. The debris sizes from the 400# and 2000# sandpapers are large (Fig. 5(a,e)), those from the 1200# and 1500# sandpapers are small (Fig. 5(c,d)), and that from the 600# sandpaper decreased the most (Fig. 5(b)). The debris size is uniform, but those from the 1500# sandpapers are uneven (Fig. 5(d)).

**Figure 6:**
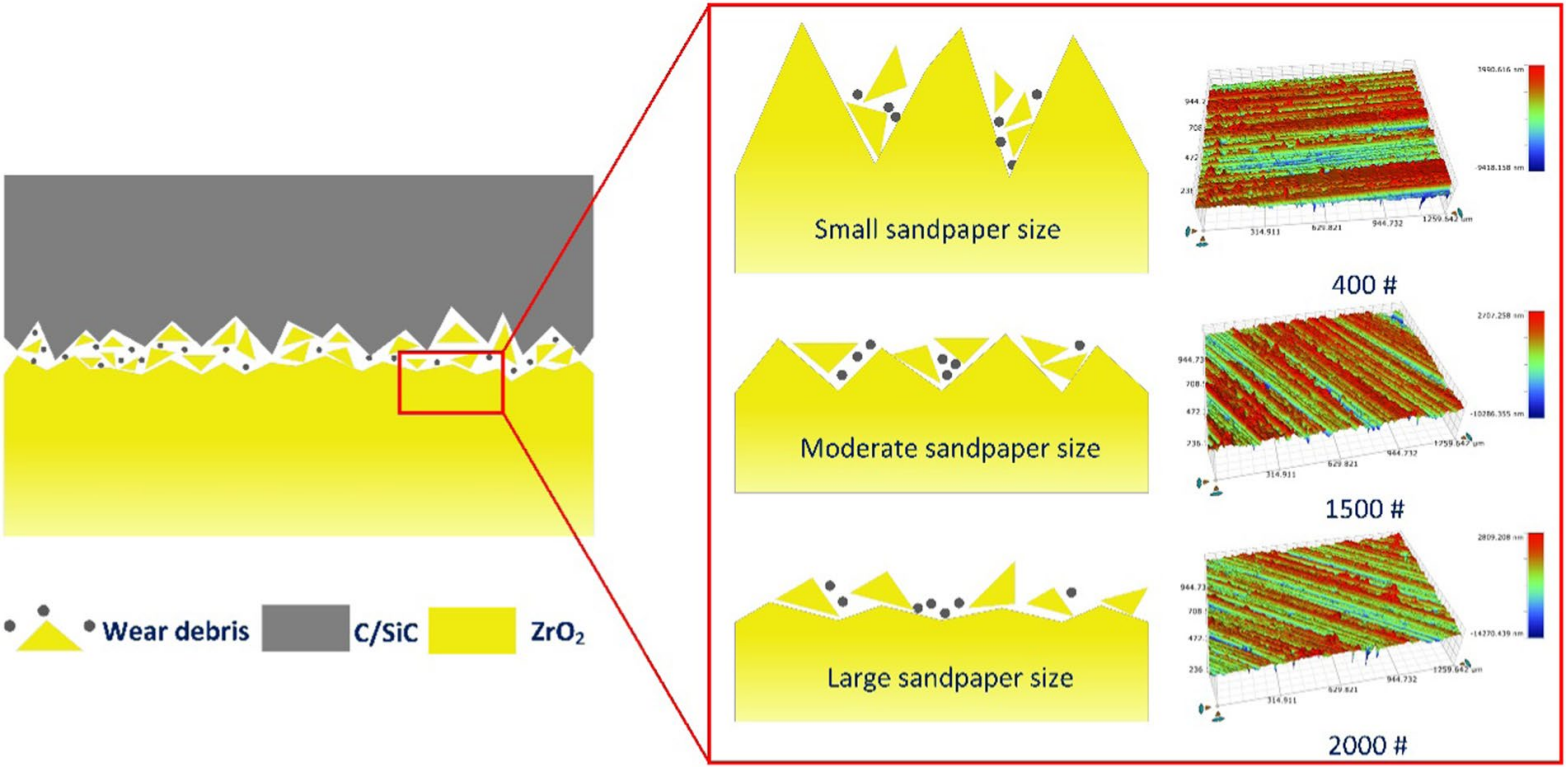
Schematic diagram of the wear mechanism.

A schematic diagram of the wear mechanism and typical wear surface 3D topography of ZrO_2_ is shown in Fig. 6. When the sandpaper number is small, the grit size is large; thus, the C/SiC pin surface has severe scratches from the grinding that act as cutting tools and grind the ZrO_2_ disc. The smaller the sandpaper grit size is, the stronger the cutting ability of the pin to the disc is. Thus, the disc surface appears to have severe friction scratches. However, the disc scratches are so wide that the wear debris retained in the scratches are constantly removed, and new debris are generated, such as for the 400# sandpaper. Therefore, the smaller the sandpaper size is, the weaker the retention ability of a disc to the wear debris.

In contrast, the larger the sandpaper size is, the weaker the cutting ability of the pin to the disc. Thus, the width and depth of the friction scratches on the disc surface decreases, and the retention ability of the disc to the wear debris decreases because the debris are unable to embed, such as for the 2000# sandpaper. The wear debris are removed quickly, and there is not much debris involved in the friction process. Therefore, the debris sizes from the 400# and 2000# sandpapers are large, and the embedded film is thin.

For the 600# sandpaper, some debris are removed from the friction scratches and, some are crushed and influence the friction because the cutting abilities of the pin to both the disc and to the debris are stronger than those of the 1200# and 1500# sandpapers. Therefore, the debris size from the 600# sandpaper is smallest. When the debris are crushed into a small size, some are retained in the disc surface. When the sandpaper size is 1500#, the disc scratches are moderate; thus, the disc can hold large parts of the debris, so the debris size increases, and some are uneven. The size of the scratches from the 1200# sandpaper is between the two situations above. The order of the cutting ability of the pin to the disc is 400# > 600# > 1200# > 1500# > 2000#, and the order of the retention ability of the disc to wear debris is 1500# > 1200# > 600# > 400# = 2000#. With a better retention ability, the debris could be hold in scratches on wear surface and reduce the third body friction. Therefore, the disc surfaces from the 600# and 1200# sandpapers have a better anti-wear and friction reduction properties than that of the surfaces from the 400# and 2000# sandpapers, and the surface from the 1500# sandpaper exhibits the best tribological performance, which corresponds to the results of Fig. 4.

## Conclusions

In this paper, the influence of the surface processing parameters of the C/SiC pin during the friction process on the tribological performance of the counterpart C/SiC-ZrO_2_ are studied. The surfaces of the pin and disc before and after the friction process are studied, and the wear mechanism is analyzed.

The counterpart of the C/SiC pin surface ground by a 1500# sandpaper and the ZrO_2_ disc ground by a diamond wheel has the best tribological performance. The Ave. COF, Max COF and SCOF are the smallest, and the initial run-in period is the shortest, among the samples studied herein. The wear rates of pin and disc present a nearly downward trend with increasing sandpaper size, but their values change slightly. Based on the characterization and analysis of wear surface and wear debris, it is found that the surface retention ability of the disc influences the tribological behavior significantly. With good surface retention ability and surface roughness, wear debris could be hold by the scratches on wear surface and reduce the third-body friction.

## Methods

The C/SiC is manufactured with chemical vapor infiltration (CVI) combined with a liquid melt infiltration process (LMI). The density of the C/SiC is 1.85 g/cm^3^. The surfaces with a 90 deg fiber orientation are processed into pins with a size of Φ4.8 mm × 12.7 mm. To research the effect of the C/SiC surface processing parameters, the pin surfaces are ground using 400#, 600#, 1200#, 1500# and 2000# sandpapers. The friction experiments are implemented under a constant force of 30 N, sliding speed of 0.5 m/s and sliding time of 7200 s on the MMW-1 standard test rig. The counterpart disc is ZrO_2_ toughened by MgO that is customized into a size of Φ55 mm × 10 mm. The density of the ZrO_2_ is 5.64 g/cm^3^. The disc surfaces are directly ground using a diamond grinding wheel under appropriate and constant processing parameters to guarantee the surface quality and consistency.

Scanning electron microscopy (SEM) images and surface energy dispersive spectroscopy (EDS) results of the pin and disc before and after friction are analyzed to reveal the wear mechanism. The ZrO2 disc were treated with gold before SEM examination for improving the electric conductivity. The X-ray diffraction (XRD) patterns of the ZrO_2_ (5 ≤ °2θ ≤ 75) were recorded using a Panalytical Empyrean X-ray diffractometer equipped with a Cu Kα radiation source; the samples were scanned using a 2θ step size of 0.02 at 25 °C and a scanning speed of 2 °/min.

## Supplementary information

Supplementary Information.
