# Supplementary material for: Effects of the surface processing on the tribological performance of C/SiCs under dry friction
Source: Sci Rep. 2020 Apr 6;10:5990. doi: 10.1038/s41598-020-62914-y (PMC7136218; doi:10.1038/s41598-020-62914-y)
Supplement: Supplementary file 1 — Supplementary Information. [file 41598_2020_62914_MOESM1_ESM.docx]

**Effects of the surface processing on the tribological performance of C/SiCs under dry friction**

Bin Lin^a,1^, Jinhua Wei^a,2^, Tianyi Sui^a,*^, Haoji Wang^a,3^

* Corresponding Author. (Tianyi Sui) E-mail: suity@tju.edu.cn

^1^ linbin@tju.edu.cn, ^2^15222527210@163.com, ^3^18822408206@163.com,

^a^ Key Laboratory of Advanced Ceramics and Machining Technology of Ministry of Education, Tianjin University, Tianjin, China

**1. The wear debris sizes of different sandpapers**

**
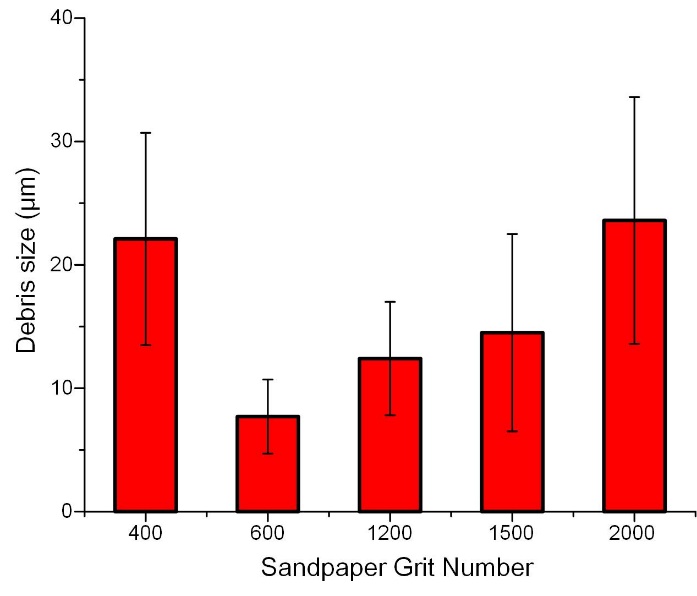
**

Fig. S1 The statistical results of the wear debris for different tribology test
